# On Preventing the Extinction of the Physician-Scientist in Pediatric Pulmonology

**DOI:** 10.3389/fped.2014.00004

**Published:** 2014-01-21

**Authors:** Ronald C. Rubenstein, James L. Kreindler

**Affiliations:** ^1^Division of Pulmonary Medicine, Cystic Fibrosis Center, The Children’s Hospital of Philadelphia, Philadelphia, PA, USA; ^2^Department of Pediatrics, Perelman School of Medicine, University of Pennsylvania, Philadelphia, PA, USA

**Keywords:** pediatrics, pulmonology, physician-scientist, training, career development

## Abstract

While the founders of Pediatric Pulmonology recognized the necessity of research as a vital part of the developing sub specialty, the field has struggled to develop and maintain physician-scientists and investigators. The clinical growth in Pediatric Pulmonology has resulted in significant challenges in career development faced by physician-scientists who aim to establish or maintain independent investigative programs. Such challenges may only be overcome with changes in how both trainees and established physician-scientists in Pediatric Pulmonology are supported.

## Introduction

The genesis of Pediatric Pulmonology as an independent, board recognized subspecialty can be traced to the late nineteenth and early twentieth centuries (1). During that time, there were coalescing interests of pioneers in the lung physiology in children, the aberrant physiology of the lungs of pre-mature infants, and the pathological states of diseases like Cystic Fibrosis and Tuberculosis. By the mid twentieth century, informal and formal associations of like-minded physicians in both the United States and in Europe (1, 2) were beginning to define what would eventually be recognized as Pediatric Pulmonology. In the United States, despite the significant burden of respiratory disease in pediatric patients, the formalization of Pediatric Pulmonology as a board recognized subspecialty was not a unanimously supported development (3). Overcoming early resistance, a group of dedicated pediatricians led by Drs. Edwin Kendig, Warren Waring, and Lynne Taussig, as well as other influential pioneers, was ultimately successful in establishing the Pediatric Pulmonology sub-board of the American Board of Pediatrics in 1985. The importance of research was tantamount to this group in the creation of Pediatric Pulmonology and is best exemplified by the words of the late Dr. Robert Mellins in a letter to the American Board of Pediatrics (3). Dr. Mellins wrote in 1983:

“…while one cannot legislate academic careers, programs are much more likely to result in academic chest physicians if there is a serious commitment to pursuing research early on and that three years of training seems minimal. Indeed, most successful academicians have had several years more of training as junior faculty beyond the fellowship year…. Nothing is as likely to be counterproductive to the maintenance of high standards as watering down the requirements.” (3)

It is with great appreciation for this hard-fought and research-focused beginning that we approach the current state of Pediatric Pulmonology a brief 30 years later. In many ways, the vision of the field’s founders has been realized by a vigorous, international coalition of Pediatric Pulmonologists making important contributions to both basic and clinical research. Moreover, the current president-elect of the American Thoracic Society, Dr. Thomas Ferkol is a Pediatric Pulmonologist, marking just the second time in the Society’s more than 100 years of existence that the society will be led by a member of this field (Dr. Mellins held the position from 1982 to 1983). Despite these positive developments, the academic future of Pediatric Pulmonology faces significant challenges presented by both internal and external forces. Here, we discuss some of these challenges in the hopes of stimulating conversation around how they can be met and overcome to carry the subspecialty forward.

## The Challenge of Clinical Growth and Expansion

One of the early concerns regarding Pediatric Pulmonology was that it overlapped other subspecialties – Allergy and Immunology, Neonatology, Critical Care – too closely to warrant being its own entity. On the contrary, understanding the normal and abnormal physiology and care of children with respiratory disorders has since been bolstered by important, physician-driven technological advances that allowed more specialized clinical and physiologic evaluation, e.g., the development of technology to measure respiratory physiology in infants and the advent of pediatric-sized flexible bronchoscopes. These and other advances have allowed Pediatric Pulmonology to expand its scope significantly over time. For example, the Pediatric Pulmonologist’s interest in the physiology of the breathing child has led to the rapidly growing field of Pediatric Sleep Medicine, which arose from within Pediatric Pulmonology and has produced many of the current leaders in the field.

Children with sleep-disordered breathing are only one example of the growing population of children who regularly see Pediatric Pulmonologists. Pediatric Pulmonologists are also regularly called upon to care for children with neuromuscular weakness, neurologic dysfunction, or chest wall abnormalities as a cause of abnormal physiology of breathing. Many of these children, including graduates of neonatal intensive care and children with complicated congenital heart disease require long-term support of technology, including mechanical ventilators, as part of their ongoing care. This too, as well as care of patients with cystic fibrosis and more common – yet still challenging – chronic disorders like asthma, has become part of the ever-expanding purview of the Pediatric Pulmonologist.

Perhaps because of the early organization of specialized multidisciplinary care centers by the Cystic Fibrosis Foundation in the early 1960s, many Pediatric Pulmonary Centers have adopted this multidisciplinary approach to care of children with chronic respiratory disease. While the time and effort of care providers such as physical therapists, nutritionists, nurses, nurse practitioners, and social workers are partially supported throughout the CF care network through care center grants from the CF Foundation, this labor and resource intensive care model is often challenged in other areas by the realities of demand and finance. In many cases, this leads to growth of highly specialized clinical programs without parallel growth of a supportive infrastructure. The end result potentially limits the flexibility of the academic physician to perform research and scholarly pursuit due to expanding clinical duties and responsibilities. In this sense, the successful expansion of clinical Pediatric Pulmonology has had the unintended consequence of making research efforts more difficult, and puts at risk the ability of the field to develop fundamental new knowledge that will ultimately benefit children with lung disease.

## The Challenge of Creating the Pediatric Pulmonologist/Physician-Scientist in Today’s Academic Environment

While certainly not the only field of investigative import in Pediatric Pulmonology, noticeably scarce within the specialty is a critical mass of pediatric physician-scientists with interests in the cellular and molecular biology underlying pulmonary and respiratory disorders. It is such physician-scientists that may be most at risk for extinction within the specialty. Historically, budding physician-scientists in Pediatric Pulmonology were often directed to and mentored by basic scientists, including MDs, PhDs, and MD/PhDs, in either the larger and better established adult pulmonary divisions at their academic centers or to scientists in related areas. These areas included renal epithelial physiology for those interested in epithelial ion transport in CF, or microbiology for those interested in lung infections. One example of this is the long-standing efforts of the CF Foundation to recruit scientists from outside the field to CF focused research. Because of their investigative work, many of these scientists became *de facto* leaders in CF basic research, especially with regards to epithelial biology and microbiology, as well as collaborators to investigators in Pediatric Pulmonology.

The traditional pathway for academic development proceeds from Pediatric residency to subspecialty fellowship, during which a physician establishes an academic focus that forms the basis for career development. In order for a field such as Pediatric Pulmonology to meet the increasing clinical needs described above and to produce thought leaders, two criteria must be met. First, there needs to be steady growth of the specialty’s ranks, which means more Pediatric residents entering into the field. Second, there need to be adequate numbers of specialists to serve the clinical needs of the at-risk population base. Said another way, the health of an academic field, in this case Pediatric Pulmonology can be assessed by examining the balance of its retiring (or aging) workforce and its incoming workforce with the expansion of the population for which the specialty cares.

### Workforce considerations

From this perspective, Pediatric Pulmonology is treading water, at best. According to ABP workforce surveys and data collection, the average age of the 1,091 board certified Pediatric Pulmonologists in the United States in 2012 (the last year for which data are available) was 52.5 years. Not only is this relatively small workforce aging, but also only 16.8% of diplomates were 31–40 years of age compared with 20.5% of diplomates being 61 years of age or older. This suggests that if current trends persist, the number of board certified Pediatric Pulmonologists in the United States would decrease over time, even as the number of patients requiring the care of a Pediatric Pulmonologist increases.

**Figure 1:**
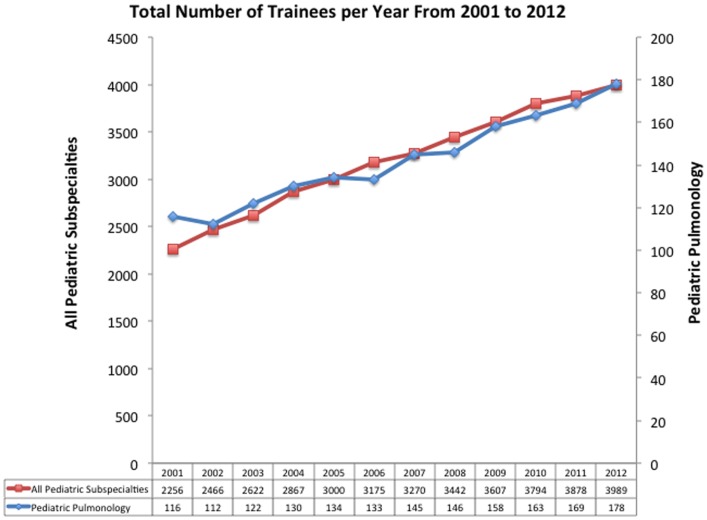
**Total numbers of trainees by year in all pediatric subspecialties and in pediatric pulmonology**. Adapted from data published by the American Board of Pediatrics.

Over the past two decades, growth of Pediatric Pulmonology has closely tracked with growth of Pediatric Subspecialists overall (Figure 1), which means that the percentage of Pediatric residents choosing Pediatric Pulmonology as a career has stayed relatively constant around 4.5%. In 2012, there were 178 Pediatric Pulmonologists in training – 65 in year 1, 57 in year 2, and 56 in year 3. The number of applicants per available fellowship position has also remained constant at 0.7 applicants per position over the last 3 years. All together, these data suggest that the number of Pediatric Pulmonologists will decline over time, especially in relation to clinical demand. In addition to the absolute number of Pediatric Pulmonologists, it is critical to understand current trends in career choice. In 2012, only two-thirds of first-time applicants for board examination expected to work full-time in an academic setting. This will even further decrease the ability of academic Pediatric Pulmonology to sustain itself.

### Training constraints

There are both internal and external factors contributing to the problems facing Pediatric Pulmonology, and these factors often coincide to exacerbate each other. For example, the current duration of fellowship training in Pediatric Pulmonology is 3 years, and most fellows enter training with interest in clinical Pediatric Pulmonology, but minimal if any prior or sustained experience in research. The first year of training at most training programs is heavily clinical in nature, thereby further delaying trainees from getting their first sustained experience in a research setting, whether clinical or basic science, until during their second year of training. Thus, by the time a trainee is fully integrated into a laboratory or clinical research program, he has an identified research focus, and begins to acquire the tools to generate and test hypotheses, it is often well into the second year or even third year of training. This makes the satisfactory completion of an independent project, which is often defined as a first author, peer-reviewed publication, by the end of 3 years of fellowship extremely difficult. Even for a trainee entering Pediatric Pulmonology fellowship with significant research experience and technical expertise, such as may have come with an advanced doctoral degree, initiating a novel project and obtaining sufficient data for publication within the time constraints of fellowship training is a challenging task. Nonetheless, in the current academic environment, achieving this difficult milestone is critical to initiation of an academic career that includes a path toward independence as a physician-scientist.

### Fellow to faculty transition

The most common pathway to an independent, academic career for subspecialty fellows is transition from fellowship to junior faculty by way of a mentored physician-scientist award, usually in the NIH K series. The competition here is steep, with only 14% of the 77 NHLBI K08 awards between 2007 and 2010 being made to Pediatricians (4). According to the same data, a group of physicians that received 21% of these awards (approximately 16 awards total) included internal medicine and emergency medicine physicians, pulmonologist, veterinary medicine physicians, pharmacists, nephrologists, endocrinologists, and pathologists, in addition to Pediatric Pulmonologists. Together, these data suggest that Pediatric Pulmonologists were awarded less than one K award per year during 2007–2010. There is also the possibility of obtaining an award from a private foundation or society such as the Cystic Fibrosis Foundation, American Heart Association or the American Thoracic Society, though these awards are extremely limited in number for Pediatric Pulmonologists, especially if the applicant’s interests lie outside those of the sponsoring foundation. Obtaining such a mentored career development award requires a number of important and significant prerequisites, which have been eloquently outlined in a recent editorial by Houser (5) and will be discussed here in the context of the field of Pediatric Pulmonology.

## Factors Intrinsic to the Pediatric Pulmonology Fellow

First among the pre-requisites for obtaining a mentored career development award, the budding physician-scientist in Pediatric Pulmonology must have a track record that suggests an aptitude for investigation and must have demonstrated some success. For the minority of trainees in Pediatric Pulmonology who had significant and sustained research experience prior to fellowship, like that obtained in graduate school, this track record is more easily demonstrated. Nonetheless, all budding physician-scientists must demonstrate aptitude and initial success in their new focus within Pediatric Pulmonology, which arguably equates with achieving the difficult milestone of a first authored, peer-reviewed publication (preferably in a high impact journal) based on their fellowship research. Interestingly, such a first authored, peer-reviewed publication was formerly a requirement for successful completion of a Pediatric Pulmonary Fellowship and eligibility for sub-board certification. This requirement was more recently modified in 2004 to require demonstration of scholarly activity; this scholarly activity can be demonstrated in ways other than a first authored, peer-reviewed publication (www.abp.org/abpwebsite/publicat/trainingrequirements.pdf). It is important to note that review articles and case reports should not be considered to contribute significantly to this developing track record unless coupled with and relevant to the fellow’s research findings. In contrast, abstracts and invited presentations at national or international meetings, especially if the data presented add to those already published by the trainee, do further help to demonstrate initial success and an “upward trajectory.”

Ideally, the fellowship research leads to completing the second pre-requisite that an applicant for a mentored career development award has a well-defined project and area of investigation that has a high likelihood of success. Here again, demonstrating such a project requires the aforementioned publication of a peer-reviewed paper in a high impact journal, and perhaps abstracts or presentations with additional data, as a starting point. But the likelihood of success of the project is not confined to simply publications based on the proposed work. In this career development context, the potential success of a project is also defined as obtaining a data set that would reasonably be expected to support future applications for independent (R01 type) funding, as well as developing the experimental and analytical tools needed to develop and test independent and novel hypotheses.

A cogent plan for how the budding physician-scientist will obtain the background knowledge and analytical tools to foster career development is a key aspect of eventual independent success. Since most who aspire to this career path have not studied the relevant biology or analytic techniques in depth, additional focused and targeted study at a graduate level is required. Recognition of gaps in previous training and a well-defined plan to fill in these gaps with graduate or specialized didactic work to complement the research training is, therefore, a third pre-requisite along the path to independence.

Common to achieving these pre-requisites is the importance of adequate, mentored time to focus on the chosen research area. However, as pointed out above, trainees are often just establishing their investigative momentum at the beginning of their third year of training at exactly the moment they would be expected to apply for the career development award that will fund their junior faculty efforts. Furthermore, at the completion of the third year of training, these newly minted boards eligible Pediatric Pulmonologists are able to independently perform clinical duties and, as a result of the high clinical demand discussed above, become highly sought after for clinical faculty positions. That these clinical positions often come with significant increase in financial compensation increases their attractiveness and decreases the attractiveness of the challenges in further developing as a physician-scientist. However, such entry clinical positions come at the cost of essentially extinguishing future investigative ability, as most newly minted Pediatric Pulmonologists have, at best, rudimentary investigative skills obtained during the 3 years of fellowship training, and essentially no free time to pursue investigation.

Several opportunities might be considered to bolster research training. Some fellowship programs allow for more sustained research experiences during the first year of training. While this obviously comes at the expense of clinical experience, there is no doubt that it increases the chance of achieving the difficult first major milestone of a high impact first authored publication. Similarly, as implied by the words of Dr. Mellins above, there is a need for adequate, mentored time immediately after completion of fellowship during the first years of faculty appointment. Thus, another, and not mutually exclusive, possible solution to this severe time limitation is to encourage trainees to participate in fourth and perhaps fifth years of “fellowship” that are largely dedicated to research efforts focused on obtaining funding for a faculty position. In fact, a majority of successful applicants for K08 funding have completed their fellowship training 1–2 years prior to their successful application with more than one-third of applicants receiving their award more than 9 years after completing their terminal degrees (6). However, again, this prolongation of “fellowship” path is less attractive than a transition after 3 years of fellowship because of the significant increase in salary that accompanies a faculty position, as well as the ready availability of clinical positions for graduating fellows.

## Factors Extrinsic to the Pediatric Pulmonology Fellow

The fourth major pre-requisite for the successful development of a physician-scientist in Pediatric Pulmonology is that the budding physician-scientist must have an established mentor with enough resources to support in large part the applicant’s research and training. Here, we note that established and resources are words that require operational definitions that may differ depending on the candidate and the research environment. Such an established mentor will have a track record of consistent publication of high quality original research in readily recognized journals. These mentors will preferably also have a record of past trainees who have remained in academic positions, achieved promotion and leadership roles, and who have themselves published original research. This requires significant foresight on the mentor’s part in the sense that there must be an articulated plan early on for the trainee’s research to evolve separately and independently of that of the mentor.

The mentor’s resources must include both the dollars necessary to support the actual research, typically from NIH R01 grant funding, the appropriate environment (space, equipment and, if needed collaborators) to perform the research, and most importantly the time necessary to devote to working with the fellow. Unfortunately, it is readily inferred from the numbers discussed above that established research mentors fitting this description are in significantly limited supply in Pediatric Pulmonology. This problem has become more acute over recent years with the budgetary limitations of the NIH and the loss of previously funded and productive physician-scientists in Pediatric Pulmonology. In addition, increasing clinical demand and financial pressures further limit the important ability of mentors to have the (usually unfunded) time necessary to appropriately supervise and help to develop the young physician-scientist’s project and skills.

The fifth and final pre-requisite is commitment of the institution at which the trainee resides. From the discussion above, it is clear that without appropriate protected time for *both the trainee and the mentor*, development of the investigative focus and skill set necessary to begin and potentially sustain a career as a physician-scientist is essentially a non-starter. Thus, to select promising physician-scientists and commit the appropriate financial resources to their development, institutions also quite reasonably insist on evidence during early training that may portend success in later development. Again this early success is best demonstrated by the initial publication milestone discussed above, but is often strongly supported by obtaining individual grants to support fellowship training, such as those of the NIH F series (NRSA) or fellowship training awards from private foundations such as the CF Foundation or the American Heart Association.

Some have recently suggested that one mechanism to increase support for Pediatric Pulmonary physician-scientist development is expanded use of the NIH T32 training grant mechanism. These authors further suggested the selection of “some programs as designated NIH Research Fellowship Programs … specially designed to offer the best possible training for clinician-scientists and attract pediatric pulmonology …. Fellows who show a strong interest in or aptitude for research (7).” The feasibility of this approach in light of more recent NIH funding constraints and sequester is not clear. Furthermore, in our opinion, while clearly beneficial to the Pediatric Pulmonology fellowship to provide support for training, having a “slot” on a T32 training grant either within or outside of the program may be less beneficial to the trainee, as this demonstrates less individual achievement than obtaining an independent grant. Moreover, such training grants (T32, as well as F series/NRSA) are distinctly uncommon in Pediatric Pulmonology because the track records of mentors and trainees in Pediatric Pulmonology training programs don’t compare well with other longer standing training programs.

To panels who review applications for mentored physician-scientist grants, there is a clear ethos that “if the institution that knows this person better than we do won’t strongly commit to this applicant, then why should we?” In essence, institutional commitment is the ultimate statement of confidence, and the strongest statements of confidence include appointment as faculty (with commensurate salary and opportunity for advancement), guarantees of protected time and the necessary resources to perform the research, including purchase of supplies, independent laboratory or work space, and appropriate technical or coordinator support personnel. For institutions with “tenure clocks” where timing of initial faculty appointment is critical in portending future promotion, these issues should be clearly explained in the statement of institutional commitment.

## The Endangered Independent Physician-Scientist in Pediatric Pulmonology

Even with strong institutional commitment and successful competition for a mentored career development award, the budding Physician-Scientist in Pediatric Pulmonology is not assured of continued success. In fact, according to data from 2000 to 2005, only about half of holders of mentored career development (K01, K08, or K23) awards were successful in obtaining subsequent NIH funding, with only some (50–60%) of these being R01s; the remainder either had unsuccessful applications or did not apply for additional NIH funding (6). Furthermore, those applying for NIH funding without the benefit of a K award were even less successful. These sobering numbers, in addition to those discussed above, suggest that creation of a new physician-scientist in Pediatric Pulmonology will not be common, and may not even replace the attrition of those retiring or ceasing to perform research because of decreasing NIH funding and increasing competition for that funding.

The physician-scientist who can translate research findings into better prevention, therapy, and outcomes remains valued, at least nominally, as evidenced by the continued existence of Medical Scientist Training Programs, or combined MD/graduate degree programs, as well as the continued existence of mentored physician-scientist awards of the K series. However, these mechanisms alone have not been sufficient to establish a critical mass of productive physician-scientists in the relatively young and undersubscribed field of Pediatric Pulmonology. If the endangered physician-scientist in Pediatric Pulmonology is valued as a means to drive the field forward and improve our ability to care for children with pulmonary disease, preventing the extinction of the species will require additional support and directed action. The current system of reliance on primarily extramural support appears to be inadequate to meet the growing needs of the field; a change in the system is necessary. This change likely means greater institutional investment in training academic Pediatric Pulmonologists during and immediately after fellowship.

Efforts must be made to maintain the small cadre of established physician-scientists in Pediatric Pulmonology who can inspire and train new physician-scientists in the field. In the present day, when a single R01 grant is often insufficient means to maintain a vigorous and productive investigative program that can also serve as a fostering environment for trainee development, additional financial support is essential. Such additional financial support is also critical to maintain momentum and productivity through multiple cycles of applying and revising applications for grant support. Institutions must resist assigning additional clinical responsibilities just to meet the ever increasing clinical need, as these break momentum and inhibit both research productivity and the ability to mentor.

Similarly, institutions must find ways to maximize the newly bred physician-scientist’s chance to grow into someone who, in turn, can inspire and train the next generation. Earlier initiation of and sustenance of protected and productive research effort appears to be the critical factor in potentially becoming a physician-scientist. Without this, the likelihood of the opportunity to continue along this path after fellowship training becomes unlikely.

Interestingly, while these suggestions, or requirements, to work toward preventing the extinction of physician-scientists in Pediatric Pulmonology may seem like a philosophical change in the field to some, they seem entirely consistent with the statements of Dr. Mellins at the birth of Pediatric Pulmonology a mere 30 years ago.

## Conflict of Interest Statement

The authors declare that the research was conducted in the absence of any commercial or financial relationships that could be construed as a potential conflict of interest.
